# Transcriptional analysis highlights three distinct immune profiles of high-risk oral epithelial dysplasia

**DOI:** 10.3389/fimmu.2022.954567

**Published:** 2022-09-02

**Authors:** Chai Phei Gan, Bernard Kok Bang Lee, Shin Hin Lau, Thomas George Kallarakkal, Zuraiza Mohamad Zaini, Bryan Kit Weng Lye, Rosnah Binti Zain, Hans Prakash Sathasivam, Joe Poh Sheng Yeong, Natalia Savelyeva, Gareth Thomas, Christian H. Ottensmeier, Hany Ariffin, Sok Ching Cheong, Kue Peng Lim

**Affiliations:** ^1^ Cancer Immunology and Immunotherapy Unit, Cancer Research Malaysia, Subang Jaya, Malaysia; ^2^ Department of Paediatrics, Faculty of Medicine, University of Malaya, Kuala Lumpur, Malaysia; ^3^ Cancer Research Center, Institute for Medical Research, National Institutes of Health, Ministry of Health Malaysia, Shah Alam, Malaysia; ^4^ Department of Oral and Maxillofacial Clinical Sciences, Faculty of Dentistry, University of Malaya, Kuala Lumpur, Malaysia; ^5^ Oral Cancer Research and Coordinating Center, Faculty of Dentistry, University of Malaya, Kuala Lumpur, Malaysia; ^6^ Faculty of Dentistry, Malaysian Allied Health Sciences Academy (MAHSA) University, Jenjarom, Malaysia; ^7^ Integrative Biology for Theranostics, Institute of Molecular Cell Biology, Agency for Science, Technology and Research (A*STAR), Singapore, Singapore; ^8^ Department of Anatomical Pathology, Singapore General Hospital, Singapore, Singapore; ^9^ Head and Neck Center, Institute of Systems, Molecular and Integrative Biology, University of Liverpool, Liverpool, United Kingdom; ^10^ Cancer Sciences, University of Southampton, Southampton, United Kingdom

**Keywords:** immune signature, non-immune reactive, immune cytotoxic, oral premalignant lesion, oral epithelial dysplasia, oral potentially malignant disorder

## Abstract

Oral potentially malignant disorders (OPMD) are precursors of oral squamous cell carcinoma (OSCC), and the presence of oral epithelial dysplasia (OED) in OPMD confers an increased risk of malignant transformation. Emerging evidence has indicated a role for the immune system in OPMD disease progression; however, the underlying immune mechanisms remain elusive. In this study, we used immune signatures established from cancer to delineate the immune profiles of moderate and severe OED, which are considered high-risk OPMD. We demonstrated that moderate and severe OEDs exhibit high lymphocyte infiltration and upregulation of genes involved in both immune surveillance (major histocompatibility complex-I, T cells, B cells and cytolytic activity) and immune suppression (immune checkpoints, T regulatory cells, and tumor-associated macrophages). Notably, we identified three distinct subtypes of moderate and severe OED: immune cytotoxic, non-cytotoxic and non-immune reactive. Active immune surveillance is present in the immune cytotoxic subtype, whereas the non-cytotoxic subtype lacks CD8 immune cytotoxic response. The non-immune reactive subtype showed upregulation of genes involved in the stromal microenvironment and cell cycle. The lack of T cell infiltration and activation in the non-immune reactive subtype is due to the dysregulation of *CTNNB1, PTEN* and *JAK2*. This work suggests that moderate and severe OED that harbor the non-cytotoxic or non-immune reactive subtype are likely to progress to cancer. Overall, we showed that distinct immune responses are present in high-risk OPMD, and revealed targetable pathways that could lead to potential new approaches for non-surgical management of OED.

## Introduction

Oral squamous cell carcinoma (OSCC) is prevalent worldwide and may be preceded by oral potentially malignant disorders (OPMD). OPMD comprises a range of oral mucosal conditions, including leukoplakia, erythroplakia, lichen planus, and oral submucosal fibrosis (1). Oral leukoplakia is the most common OPMD, with a global prevalence of 4.1% and a malignant transformation rate between 0.1%-34.0% (2–4). Notably, the malignant transformation rate increases to 40% in the presence of oral epithelial dysplasia (OED) (5). Current clinical management of OPMD depends on the severity of OED (4, 6). OPMD patients with the presence of hyperplasia or mild OED have a lower risk of malignant transformation; hence, they are usually subjected to watchful waiting. By contrast, OPMD with moderate or severe OED is surgically removed because of it’s association with a higher risk of malignant transformation. However, the current clinical approach may lead to the risk of under- or over-treatment, as low-risk OPMD may progress and high-risk OPMD may regress, underscoring the need for better risk stratification strategies and novel therapeutic interventions.

Most OPMD studies have focused on identifying genomic and molecular aberrations in the epithelial compartment (7–10). Commonly reported aberrations include loss of heterozygosity (LOH) at chromosomal sites 3p and/or 9p and DNA aneuploidy, which lead to an increased risk of malignant transformation of 6-fold and 3-fold, respectively. Others, such as *TP53, EGFR*-family members and cell cycle regulators commonly found in OSCC, have also been documented in OPMD (10). Unfortunately, these molecular alterations have yet to be translated into clinical utility because their effectiveness as biomarkers of malignant transformation and/or chemopreventive targets is limited (11).

Recent studies have demonstrated that host immunity may be a key determinant of premalignant disease outcome (12, 13). In OPMD, the accumulation of immune cells is commonly observed in dysplastic areas (14–17). Common changes reported in the immune microenvironment of OPMD are similar to other premalignant lesions, including CD3 lymphocytes, CD8 T cells, T regulatory (Treg) cells, M1/M2-macrophages and the PD-1/PD-L1 axis (12, 13, 17–26). However, knowledge on the immune profile of OPMD remains limited. In this study, we used immune signatures derived from cancer to determine the immune profiles of moderate and severe OEDs, which are considered high-risk OPMD. Our study demonstrated that although inflammatory responses are highly induced in high-risk OPMD, three distinct immune subtypes were detected, which may provide information on disease outcomes and potential targets for intercepting malignant transformation.

## Materials and methods

### Sample collection

A total of 98 oral leukoplakias were retrospectively selected and further grouped into hyperplasia (also known as non-dysplastic leukoplakia; NDL), mild OED, moderate OED, and severe OED (1). Additionally, 23 fibroepithelial polyps (FEP) and six early stage OSCC (pathological stage I/II) were included. Histopathological assessments of hematoxylin and eosin (H&E)-stained tissue sections were performed by two board-certified oral pathologists (RBZ and SHL), and formalin-fixed paraffin-embedded (FFPE) tissue blocks in excess of diagnosis were retrieved from the Faculty of Dentistry, University of Malaya, and the Stomatology Unit, National Institute of Health, Malaysia. This study was approved by the Medical Ethics Committee of the Faculty of Dentistry, University of Malaya [DF OS1624/0073(L)], and the Medical Research and Ethics Committee (MREC), Ministry of Health Malaysia [NMRR-16-1764-32566 (IIR)]. The collected specimens were used for histopathological evaluation of immune infiltrate, RNA sequencing and multiplex immunofluorescence (mIF) staining, as detailed in **Supplementary Table 1**.

### Histopathological evaluation of immune infiltration in H&E-stained tissue sections

Immune cell infiltration was examined using digital images of H&E-stained tissue sections. The regions of interest (ROI) for FEP, OPMD and OSCC were determined by oral pathologists and referred to the areas of hyperplasia, OED or tumor, respectively. Immune infiltrate at the ROI was assessed qualitatively according to the following patterns: (a) infiltrated from stroma into epithelial compartment, (b) present in the stroma, or (c) absence of immune infiltrate. In cases with heterogeneous immune infiltrate patterns, the decision was made based on the pattern that dominated ≥70% of the ROI.

### RNA sequencing library preparation, sequencing and data processing

FFPE tissues were macro-dissected to obtain the ROI comprising 70% of epithelium and its underlying stromal compartment. Tissue sections of 100µm thickness were subjected to RNA extraction using the RNeasy FFPE kit (Qiagen, Germany) following the manufacturer’s instructions. RNA quality was assessed using RNA 6000 Nano Kit and analyzed using an Agilent 2100 Bioanalyzer (Agilent Technologies, USA). RNA concentration was assayed using the Qubit RNA HS Assay Kit and read using a Qubit 2.0 fluorometer (Thermo Fisher Scientific, USA). Samples with ≥ 30% RNA fragments of >200 nucleotides (DV200) were selected, and 200ng of total RNA was subjected to sequencing library preparation using the NEBNext Ultra RNA Library Prep Kit (New England BioLabs, USA).

RNA sequencing was performed on Illumina HiSeq2500 (Illumina, USA) with a coverage of 50 million reads per sample to generate paired-end 150 nucleotide reads. The RNA sequencing data were filtered using Fastqp (v.0.3.1) and MultiQC (v.1.11) to remove low-quality sequences, adapters, and contaminants. The samples were aligned to the human reference genome GRCh38 using Bowtie2 (v.2.3.2) (27). Raw gene counts were obtained by subjecting BAM files to Rsubread (v.2.2.6) (28). Normalization was performed using edgeR (v.3.34.1) (29). Genes expressed in less than 20% of the samples were filtered out.

RNA sequencing was conducted in two batches. A pilot study (n=15) was performed to explore gene expression differences in FEP, oral leukoplakia with different grades of dysplasia, and early stage OSCC. Thirteen samples were successfully sequenced and analyzed. A second batch of RNA sequencing was performed and 43 of the 54 samples were successfully sequenced. Both datasets were combined and corrected for batch effects using ComBat (30). As low-risk OPMD was not the focus of this study, three NDL and two mild OED samples were excluded. Finally, the experimental set comprising FEP (n=14), moderate and severe OED (grouped as moderate-severe OED; n=31) and early stage OSCC (n=6) were subjected to subsequent analysis.

### Identification of gene co-expression at different stages of disease

The Uniform Manifold Approximation and Projection (UMAP) method was used to examine the sample distribution based on global gene expression (31). Subsequently, modules of genes co-expressed in FEP, moderate-severe OED and early stage OSCC were derived using WGCNA (version 1.69) (32). Soft-thresholding power was used for automatic network construction and module detection of all 18,938 genes. A matrix of adjacencies was constructed by computing Pearson correlations between all gene pairs across all samples, and the Topological Overlap Measure (TOM) was used to form the unsigned gene network. Modules were defined at medium sensitivity (deepSplit=2) and co-expressed genes were merged at a set threshold of 0.25. Thirty modules were identified, each comprising a minimum of 30 coexpressed genes. These modules were subjected to single-sample Gene Set Enrichment Analysis (ssGSEA version 10.0.3) using GenePattern (33). The Kruskal-Wallis test and Dunn’s multiple comparisons were used to identify modules that were differentially enriched across the three disease stages (*p*-value <0.05). Significant modules were categorized into three expression trends based on the median ssGSEA scores for each disease stage. Metascape was used to map the modules for each expression trend to derive the biological functions involved (34).

### Determination of the immune profile in moderate-severe OED by immune signatures enrichment analyses

We compiled 125 immune signatures reported in cancers for immune profiling of moderate-severe OED (35, 36). These immune signatures are gene sets involved in specific immune responses in cancer and are broadly classified into three categories: (a) immune cell infiltration, (b) immune cell type, and (c) other expression signatures. The enrichment of these immune signatures was evaluated by subjecting our RNA sequencing dataset (n=51) and the head and neck cancer (HNC) dataset (n=489) from The Cancer Genome Atlas (TCGA) to ssGSEA analysis as described above. The resulting ssGSEA scores were transformed into z-scores. Significant immune signatures were selected based on the Kruskal-Wallis test after adjusting for the false discovery rate (FDR) with a threshold of 0.1 computed using the Benjamini and Hochberg method. Finally, Dunn’s multiple comparison test was used to identify immune signatures that were significantly upregulated or downregulated in moderate-severe OED and OSCC compared to FEP.

### Deconvolution of immune cell type

To impute the immune cell composition in each sample, we subjected the RNA sequencing data to CIBERSORTx analysis (37). The relative fractions of the 22 immune cell types were deconvolved by applying the predefined LM22 signature gene matrix, with the run parameters set at 1000 permutations and B-mode batch correction. We determined the total lymphoid composition by calculating the aggregates of CIBERSORTx fractions of B cells, plasma cells, CD4 T cells, CD8 T cells and natural killer (NK) cells. Meanwhile, the fractions of monocytes, dendritic cells, mast cells, eosinophils, and neutrophils were aggregated to form the myeloid composition.

### Determination of the expression of immune co-stimulatory and co-inhibitory genes in moderate-severe OED

Immune co-stimulatory and co-inhibitory molecules are important immune modulators that govern T cell function. A total of 84 immune modulating genes identified in the literature were examined in this study (13, 38). The expression of these immune-modulating genes was extracted from our RNA sequencing dataset and normalized to the expression of TATA-box binding protein (*TBP*). The normality test was performed using D’Agostino and Pearson omnibus tests. The Kruskal-Wallis test was applied to determine statistical differences in FEP, moderate-severe OED and early stage OSCC, subsequently pairwise comparisons were performed using Dunn’s multiple comparison. Statistical significance was set at *p*-value < 0.05.

### Multiplex immunofluorescence (mIF) staining on T cell population

The T cell population in OPMD was further investigated using mIF staining on a subset of samples where tissue sections were available. In this experiment, 10 NDL and 27 moderate-severe OED were analyzed. NDL was used as an experimental control because it is a hyperplastic lesion with a low-risk of malignant transformation and demonstrated gene expression similar to that of FEP. The immune markers investigated in this study were CD45, CD3, CD8, FOXP3, and PD-L1. mIF staining was performed using the Opal Multiplex fIHC kit (Akoya Bioscience, Menlo Park, California, USA) as previously described (39). FFPE tissues of 4µm thickness were sectioned onto charged slides and heated at 60°C for 20 min. Tissue slides were subjected to deparaffinization, rehydration and heat-induced epitope retrieval (HIER) using a Leica Bond Max autostainer (Leica Biosystems, Melbourne) prior to endogenous peroxidase blocking (Leica Biosystems, Newcastle). Slides were incubated with the primary antibodies listed in **Supplementary Table 2**, followed by application of polymeric HRP-conjugated secondary antibodies (Leica Biosystems, Newcastle). An appropriate Opal fluorophore-conjugated TSA (Akoya Bioscience, USA) was added at 1:100 dilution. Slides were rinsed with washing buffer after each step. Following TSA deposition, the slides were subjected to HIER to strip the tissue-bound primary/secondary antibody complexes and ready for labelling of the next marker. These steps were repeated until all five markers were labelled, and finally spectral DAPI (Akoya Bioscience, USA) was added at a 1:10 dilution. The slides were mounted in ProLong Diamond Antifade Mountant (Molecular Probes, Life Technologies, USA) and cured in the dark at room temperature for 24 hours. Ten images from the ROI were selected and scored by a pathologist using the inForm software (version 2.4.2; Akoya Bioscience, USA) and HALO™ (Indica Labs).

### Identification of immune subtypes using unsupervised hierarchical clustering analysis

To detect the immune subtypes that are present in moderate-severe OED, we subjected 31 moderate-severe OED cases in our RNA sequencing cohort to unsupervised hierarchical clustering analysis with the 58 immune signatures identified in the previous section. To validate this finding, we extracted the gene expression data of moderate and severe OED samples (n=10) from GSE26549 for supervised hierarchical clustering analysis using the 58 immune signatures identified above (7). Hierarchical clustering analyses were performed using the one-minus Spearman rank correlation metric on Morpheus versatile matrix visualization and analysis software (https://software.broadinstitute.org/morpheus).

### Identification of oncogenic gene set enrichment in non-immune reactive subtype

To investigate whether oncogenic signals from the epithelial compartment influenced the local immune response in the non-immune reactive subtype, ssGSEA was performed using the C6 oncogenic signature gene set obtained from MSigDB v7.4. Significantly enriched oncogenic signature gene sets in the non-immune reactive and immune cytotoxic subtypes were identified based on the Mann-Whitney test after adjusting for FDR with a threshold set at 0.1. An enrichment plot was generated using the ImageGP online data visualization tool (40).

### Statistical analysis

Statistical analyses were performed using GraphPad Prism 9.1.2 (GraphPad Software, San Diego, California, USA). Descriptive statistics were used to summarize patient demographics. The chi-square test was used to analyze the pattern of immune infiltrates and sample type. Differences in immune cell populations between NDL and moderate-severe OED were calculated using unpaired *t*-tests. The Kruskal-Wallis test was used to determine differences in lymphoid compartment, immune co-stimulatory, and immune co-inhibitory molecules between FEP, moderate-severe OED and OSCC. *Post hoc* analysis was performed using Dunn’s multiple comparison test. Statistical significance was defined as *p*<0.05.

Statistical methods used in bioinformatics analyses were incorporated into the respective computer scripts and are described in the respective sections.

## Results

### Patient cohort

A total of 127 FFPE tissues consisting of 98 oral leukoplakia, 6 early stage OSCC (pathological TNM stage I/II), and 23 FEP were used in this study. Patient demographics and clinical information are summarized in **Table 1**. Two-thirds of the oral leukoplakias were at the buccal mucosa and tongue, which are common anatomical locations for oral leukoplakia globally (6). The oral leukoplakia samples consist of non-dysplastic leukoplakia (NDL, n=20) and OED (mild, n=32; moderate-severe, n=46). Disease transformation status was available for 12 moderate-severe OED patients with a follow-up duration of 2–50 months (**Supplementary Table 3**). FEP is a reactive benign hyperplastic lesion that develops because of irritation or trauma. Hence, materials from such cases were used as controls (41).

**Table 1:** Patient demographics and clinical information for cases and controls.

Sample type	FEP (n)	Oral leukoplakia (n)	OSCC (n)
**Total cases**	23	98	6
**Gender**			
Male	8	46	4
Female	15	52	2
**Age at diagnosis (years)**			
Mean	47	60	62
Range	14-86	18-99	41-84
**Ethnic**			
Malay	11	15	2
Chinese	7	22	1
Indian	5	49	2
Others	0	12	1
**Type of leukoplakia**			
Homogenous	NA	41	NA
Non-homogenous	NA	33	NA
Not specified	NA	24	NA
**Pathological diagnosis**			
FEP	23	NA	NA
NDL	NA	20	NA
Mild OED	NA	32	NA
Moderate-severe OED	NA	46	NA
OSCC T1/T2	NA	NA	6
**Site of lesion**			
Buccal mucosa	11	34	3
Tongue	2	30	3
Gingiva	2	12	0
Others	8	20	0

NA indicates not applicable.

### Moderate-severe OED showed lymphocyte infiltration into the epithelial compartment

**Figure 1:**
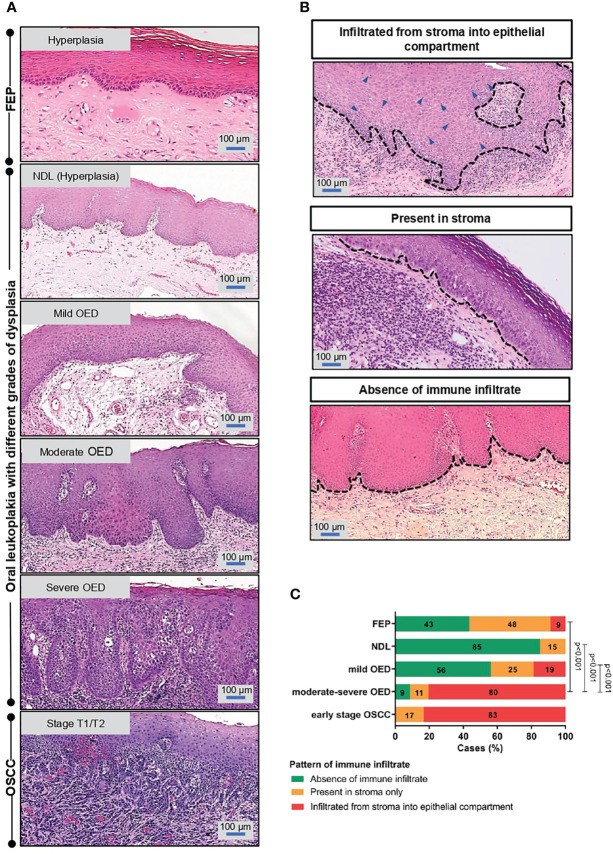
Immune infiltrate pattern is associated with the severity of OED. **(A)** Histopathological images depicting the changes of epithelial architecture from benign lesions to OSCC. Fibroepithelial polyps (FEP) is a reactive benign hyperplastic lesion. OPMD examined in this study comprised of oral leukoplakia that were histologically diagnosed with either hyperplasia (NDL), mild, moderate and severe OED. Early stage OSCC were of pathological stage T1/T2. Both FEP and NDL are hyperplastic, hence were used as control in the respective experiment. **(B)** Representative microscopic images of immune infiltrate pattern observed in H&E-stained tissue section. The pattern of immune infiltrate was graded at the region of interest (ROI) and classified into 3 groups: (i) infiltrated from stroma into epithelial compartment (ii) present in stroma (iii) absence of lymphocytic infiltrate. **(C)** A significant proportion of moderate-severe OED demonstrated immune infiltrate from stroma into the epithelial compartment. 80% of moderate-severe OED and early stage OSCC showed immune infiltrate from stroma into the epithelial compartment compared to 9% of FEP. Chi-square test was performed to determine the association of immune infiltrate pattern with disease stage.

The identified ROI for each sample type is shown in **Figure 1A**, and examples of immune infiltrate are shown in **Figure 1B**. In comparison to low-risk OPMD (NDL and mild OED), a significant proportion of moderate-severe OED (80%, n=37/46; p<0.001) and early stage OSCC (83%, n=5/6; p<0.001) demonstrated immune infiltration into the epithelial compartment (**Figure 1C**). Our histopathological findings suggest that the lymphocyte infiltration pattern is associated with OED severity.

### Immune-related genes are overexpressed in moderate-severe OED and early stage OSCC

**Figure 2:**
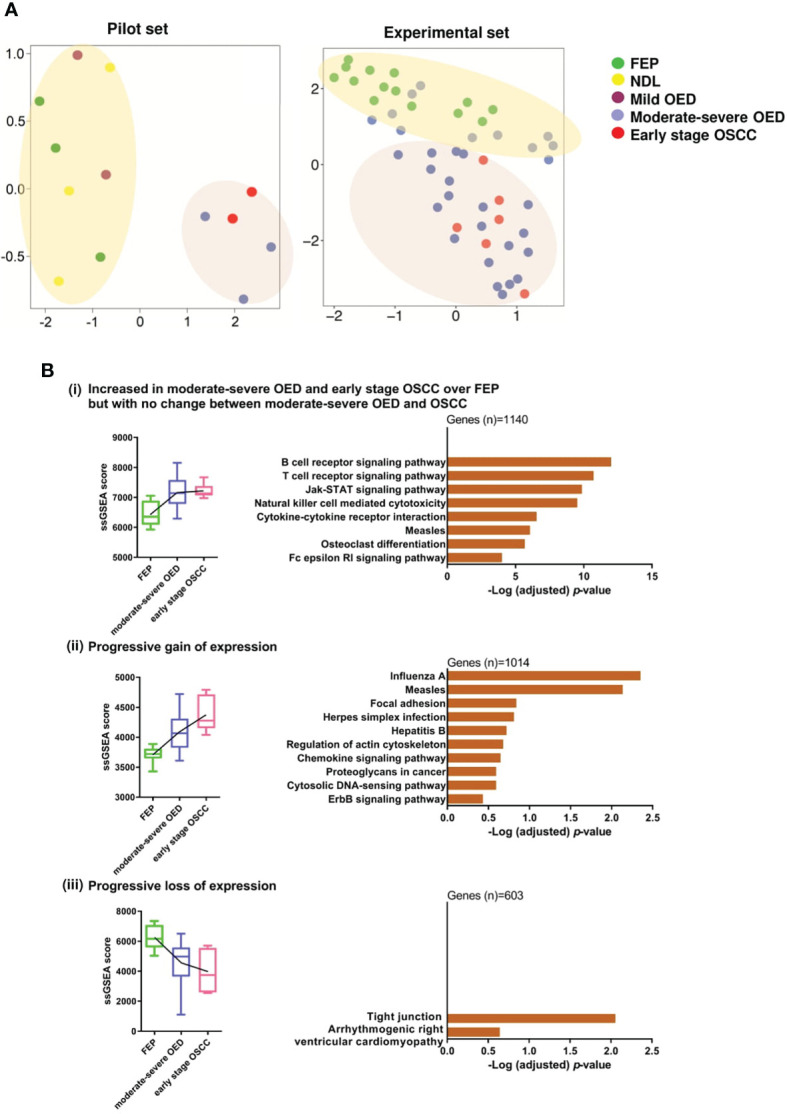
Gene co-expression analysis revealed immune-related genes are significantly overexpressed in moderate-severe OED. **(A)** Unsupervised clustering of samples based on global gene expression. UMAP plot for the pilot RNA sequencing dataset (n=13) revealed that FEP, NDL and mild OED shared a similar gene expression profile and are distinct from the moderate-severe OEDs which were grouped with early stage OSCC (left). UMAP plot of the experimental dataset (n=51) demonstrating FEP and OSCC samples formed 2 separate clusters (right). Majority of moderate-severe OED samples clustered with OSCC samples (bottom) but others were grouped with FEP samples (top). **(B)** Identification of gene co-expression trends across FEP, OED and OSCC. Pathways representing the co-expressed gene modules were shown as bar plots. (i) Increased in moderate-severe OED and early stage OSCC over FEP but with no change between moderate-severe OED and OSCC: Immune-related pathways were upregulated in moderate-severe OED and sustained at a similar level in early stage OSCC. (ii) Progressive gain of expression: A mixture of genes involved in cancer-related and immune-related pathways continuously upregulate from FEP to moderate-severe OED and further into early stage OSCC. (iii) Progressive loss of expression: Genes involved in tight junction pathway are continuously downregulated from FEP to moderate-severe OED and further in early stage OSCC.

Unsupervised clustering analysis of the pilot RNA sequencing samples (n=13) identified two clusters of samples. The first cluster contained FEP and low-risk OPMD (NDL and mild OED), while the second cluster comprised high-risk OPMD (moderate-severe OED) and early stage OSCC (**Figure 2A** **). Because the gene expression in high-risk OPMD is similar to that of early stage OSCC, we decided to focus on moderate-severe OED in the second batch of RNA sequencing. Finally, an experimental set comprising** moderate-severe OED (n=31), FEP (n=14), and early stage OSCC (n=6) was formed. Unsupervised clustering analysis performed on the experimental set showed that FEP and early stage OSCC samples formed distinct clusters. Moderate-severe OEDs overlapped across these two clusters, with 64% (n=20/31) clustered with early stage OSCC and 36% (n=11/31) clustered with FEP, indicating that most moderate-severe OED had acquired a gene expression profile similar to that of early stage OSCC.

Next, we used WGCNA to identify gene modules that were co-expressed across the three disease stages: FEP, moderate-severe OED, and early stage OSCC. Specifically, nine modules were differentially enriched in moderate-severe OED and OSCC compared to FEP (*p*<0.05; **Supplementary Figure 1**). These nine modules were grouped into three expression trends: (i) increased in moderate-severe OED and OSCC over FEP, but with no change between moderate-severe OED and OSCC (n=1140 genes), (ii) progressive gain of expression from FEP to OSCC (n=1014 genes), and (iii) progressive loss of expression from FEP to OSCC (n=603 genes). Metascape pathway analysis revealed that the genes in trend (i) are related to immune responses **(** **Figure 2B**). These include T cell and B cell receptor signaling, JAK-STAT signaling, cytokine-cytokine receptor interactions, and NK cell-mediated cytotoxicity. Genes in trend (ii) are involved in heterogeneous pathways, including immune responses (chemokine signaling and cytosolic DNA-sensing pathways), cell shape and motility (focal adhesion and regulation of the actin cytoskeleton), cancer development (proteoglycans in cancer and the ErbB signaling pathway), and viral infections. Genes in trend (iii) are involved in the tight junction pathway. The downregulation of the tight junction pathway indicates the loss of cell-cell adhesions and cellular barrier function of the epithelium, as reflected by the histological changes in cellular organization in moderate-severe OED and early stage OSCC. Overall, our gene co-expression data indicated that immune-related genes were upregulated in moderate-severe OED and sustained in early stage OSCC. However, cancer-related genes continue to be upregulated, and genes regulating cellular organization continue to be downregulated across the disease stage.

### Immune signature analyses revealed the induction of immune surveillance and immune suppressive mechanisms in moderate-severe OED

Since immune-related genes are upregulated in moderate-severe OED and early stage OSCC, we further delineated the underlying immune phenotype and function using 125 immune signatures derived from cancer (35, 36). Of the 125 evaluated immune signatures, 58 showed differential enrichment when comparing moderate-severe OED and early stage OSCC to FEP (**Supplementary Figure 2**). Specifically, 81% (47/58) of the immune signatures demonstrated increased enrichment in moderate-severe OED and/or early stage OSCC.

**Figure 3:**
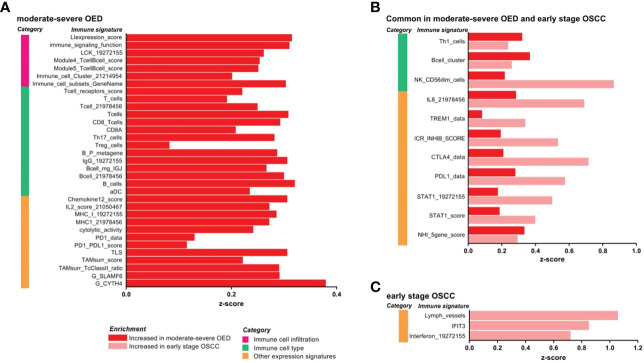
IImmune signature enrichment analysis revealed the similarity and differences of immune response underlying the immune infiltrates in moderate-severe OED and early stage OSCC. **(A)** Immune signatures enriched in moderate-severe OED. Immune signatures representing immune cell infiltration, T cells and CD8 T cells and cytotoxic response were enriched suggesting an active immunosurveillance is ongoing in moderate-severe OED. At the same time, induction of immune suppressive mechanisms such as Treg and immune checkpoints (*PD-1/PD-L1*) were detected. **(B)** Immune signatures commonly enriched in moderate-severe OED and early stage OSCC. Immune signatures for Th1, B cells and cytotoxic NK cells are commonly enriched in both moderate-severe OED and early stage OSCC. Notably, those involve in pro-inflammatory responses (IL8, TREM1 and STAT1) and immune checkpoints (ICR_INHIB_score, *PD-L1* and *CTLA4*) were further enriched in early stage OSCC compared to moderate-severe OED. **(C)** Immune signatures enriched in early stage OSCC. Immune signatures representing interferon and lymphatic vessels were enriched in early stage OSCC.

Our data revealed that both immune surveillance and immune suppressive mechanisms were induced in moderate-severe OED compared with FEP (**Figure 3A**). The immune surveillance responses are represented by immune signatures for antigen presentation (MHC-I), lymphocytes infiltration (LIexpression_score, LCK_19272155, and Module4_TcellBcell_score) and immune cytotoxic response (CD8_Tcells, and cytolytic activity). The enrichment of immune signatures representing lymphocyte infiltration was consistent with our histological assessment, which predominantly showed the presence of lymphocytes in the epithelial or stromal compartments. Moreover, the immune signatures of cytokines associated with lymphocyte proliferation were also enriched (IL2_score_21050467 and Chemokine12_score). Concomitantly, immune signatures involved in immune suppressive mechanisms, such as Treg_cells, immune checkpoints (PD-1/PD-L1) and surrogate markers of tumor-associated macrophages (TAMsurr_score), were also enriched in moderate-severe OED.

In addition, we identified immune signatures that were commonly enriched in moderate-severe OED and early stage OSCC (**Figure 3B**). Immune signatures for a subset of lymphocytes, such as Th1 cells, B cells and cytotoxic NK cells (NK_CD56dim_cells), were enriched in both disease stages. However, specific pro-inflammatory responses represented by immune signatures for *IL-8, TREM1* and *STAT1*, and those representing immune checkpoints (*PD-1, PD-L1, CTLA4* and ICR_INHIB_score) were further enriched in early stage OSCC when compared to moderate-severe OED, although not statistically significant. In early stage OSCC, immune signatures representing interferon, IFIT3 and lymphatic vessels were enriched when compared to FEP (**Figure 3C**). Given the small sample size of patients with early stage OSCC in our dataset, we further examined these immune signatures in the TCGA-HNC dataset. Importantly, the immune signatures enriched in our dataset were also recapitulated in early stage tumors in the TCGA-HNC dataset (**Supplementary Figure 3**). Moreover, the TCGA-HNC dataset indicated that enrichment of these immune signatures was maintained in late stage tumors.

### Increased total lymphoid composition in moderate-severe OED

**Figure 4:**
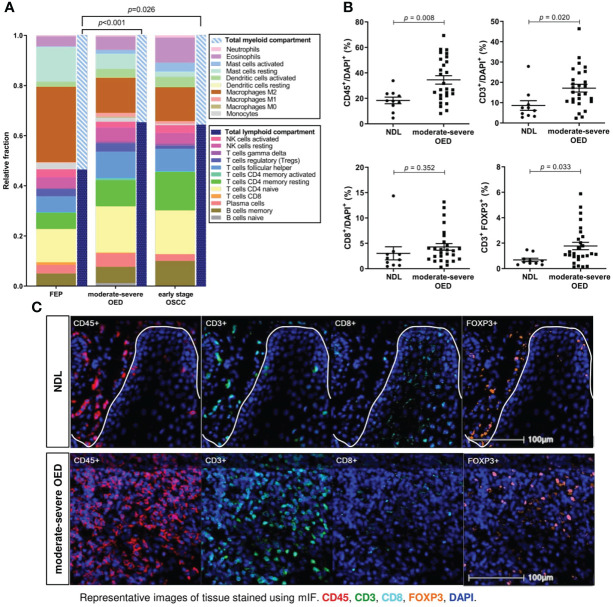
T cells are markedly increased in moderate-severe OED. **(A)** CIBERSORTx analysis estimated the increase of total lymphoid compartment in moderate-severe OED and early stage OSCC. Stacked bar chart representing the relative fractions of 22 immune cell subsets. The increase of lymphoid compartment was attributed by T cell and B cell fractions. **(B)** Validation of increased T cell population in moderate-severe OED by mIF staining. Dot plots representing the percentage of immune cells expressing the specific immune markers in each sample. Mean and standard error of mean are indicated for each group of samples. In comparison to NDL, moderate-severe OED showed significant increase of CD45^+^ leukocyte population (*p*=0.008), CD3^+^ T cell population (*p*=0.020) and FOXP3^+^ Treg population (*p*=0.015). A higher CD8^+^ cytotoxic T cell population was detected in moderate-severe OED but was not statistically significant (*p*=0.351). **(C)** Representative mIF images at the ROI of NDL and moderate-severe OED. Images shown were of 25x magnification. The white solid line indicates the epithelial-stroma border. Immune markers were labelled as following: CD3 (green), CD45 (red), CD8 (cyan), FOXP3 (orange) and DAPI (blue).

We performed CIBERSORTx analysis to deconvolve the immune cell types present in FEP, moderate-severe OED and OSCC. We observed a balanced proportion of lymphoid (T cells, B cells, plasma cells, and NK cells) and myeloid (monocytes, mast cells, DC, neutrophils, macrophages, and eosinophils) compositions in the FEP group (**Figure 4A**). An induction of adaptive immune response was observed in moderate-severe OED and early stage OSCC, as the lymphoid cell composition significantly increased when compared to FEP. Among the lymphoid cell compositions, T and B cell subsets were increased in moderate-severe OED, which was consistent with our immune signature enrichment analysis.

Bioinformatics analyses demonstrated that the T cell subset was consistently increased in moderate-severe OED. To validate this finding, we performed mIF staining on common T cell markers in moderate-severe OED and NDL. A significant increase in immune cells was observed in moderate-severe OED when compared to NDL (low-risk OPMD), specifically the populations of CD45^+^ leukocyte (34% vs. 18%; *p*=0.008) and CD3^+^ T cell (17% vs. 9%; *p*=0.020) (**Figures 4B**, **C**). However, the increase in CD8^+^ cytotoxic T cells in patients with moderatesevere OED was not statistically significant. Additionally, moderate-severe OED also demonstrated a significant increase in Treg cells compared to NDL, as indicated by the FOXP3^+^ population (5% vs. 2%; *p*=0.033). An increase in Treg cells could limit CD8^+^ T cell function.

### PD-L1 expression is increased in the immune cells of moderate-severe OED

**Figure 5:**
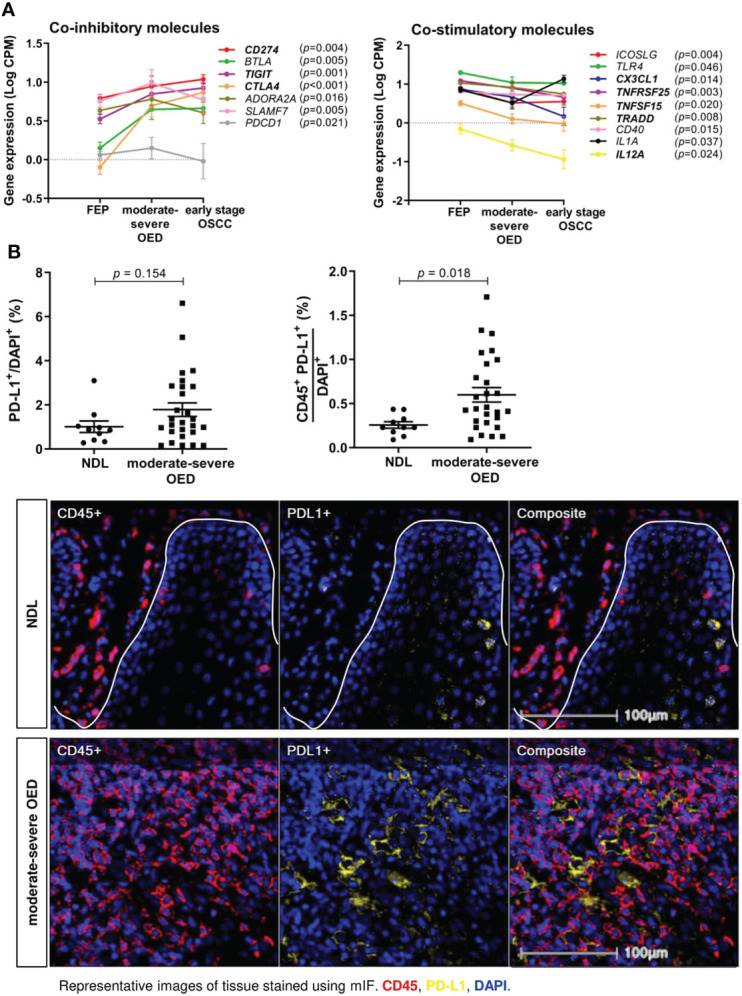
The expression of co-stimulatory and co-inhibitory molecules are altered in moderate-severe OED. **(A)** Upregulation of co-inhibitory immune molecules and downregulation of co-stimulatory immune molecules in moderate-severe OED. Line graph demonstrating the changes of mean expression of immune co-inhibitory and co-stimulatory molecules in FEP, moderate-severe OED and early stage OSCC. The error bars represent the standard error of mean. Three co-inhibitory molecules (*CD274, TIGIT* and *CTLA4*) and five co-stimulatory molecules (*TNFRSF25, TRADD, CX3CL1, TNFSF15* and *IL12A*) showed a continuous upregulation and downregulation from FEP to moderate-severe OED and early stage OSCC respectively. *CD274* and *PDCD1* encodes for PD-L1 and PD-1 respectively and is the only ligand-receptor pair found to be consistently upregulated in moderate-severe OED. **(B)** A high proportion of PD-L1-expressing immune cells detected in moderate-severe OED. A significant increase of CD45^+^PD-L1^+^ cell number rather than the total PD-L1^+^ cell number suggested that PD-L1 is expressed in the immune cells and lesser in the epithelial cells of moderate-severe OED. Mean and standard error of mean are indicated for each group of samples. Representative mIF images of DAPI, CD45 and PD-L1 staining at the ROI of NDL and moderate-severe OED. Immune markers in the images were labelled as following: CD45 (red), PD-L1 (yellow), and DAPI (blue).

Immune coinhibitory and costimulatory molecules are important drug targets in cancer. We evaluated the expression of 84 costimulatory and coinhibitory genes in the samples. Co-inhibitory molecules, including *CD274* (encoding for PD-L1), *TIGIT* and *CTLA4* were continuously upregulated from FEP to moderate-severe OED and early stage OSCC (**Figure 5A**). Meanwhile, co-stimulatory molecules involving *CX3CL1, TNFRSF25, TNFSF15, TRADD*, and *IL12A*) showed continuous downregulation from FEP to moderate-severe OED and early stage OSCC. Gradual changes of co-stimulatory and co-inhibitory molecules across FEP, moderate-severe OED and early stage OSCC, suggested that they may play a role in sustaining carcinogenesis and represent worthy targets for cancer therapy.

Notably, PD-1 and PD-L1 were the only receptor-ligand pairs that were consistently upregulated in moderate-severe OED. We conducted mIF staining on PD-L1 expression, since it is a biomarker of response to immune checkpoint blockade (ICB) treatment in cancer patients. PD-L1 expression can be detected in the epithelial and tumor-infiltrating immune cells in HNC (42). Although the total PD-L1^+^ population was not significantly higher in moderate-severe OED than in NDL, we observed a significant increase in CD45^+^PD-L1^+^ population in moderate-severe OED (0.6% vs. 0.3%; *p* = 0.018), suggesting that the immune cells in high-risk OPMD express PD-L1 (**Figure 5B**).

### Moderate-severe OED can be classified into 3 distinct subtypes by immune profiling

**Figure 6:**
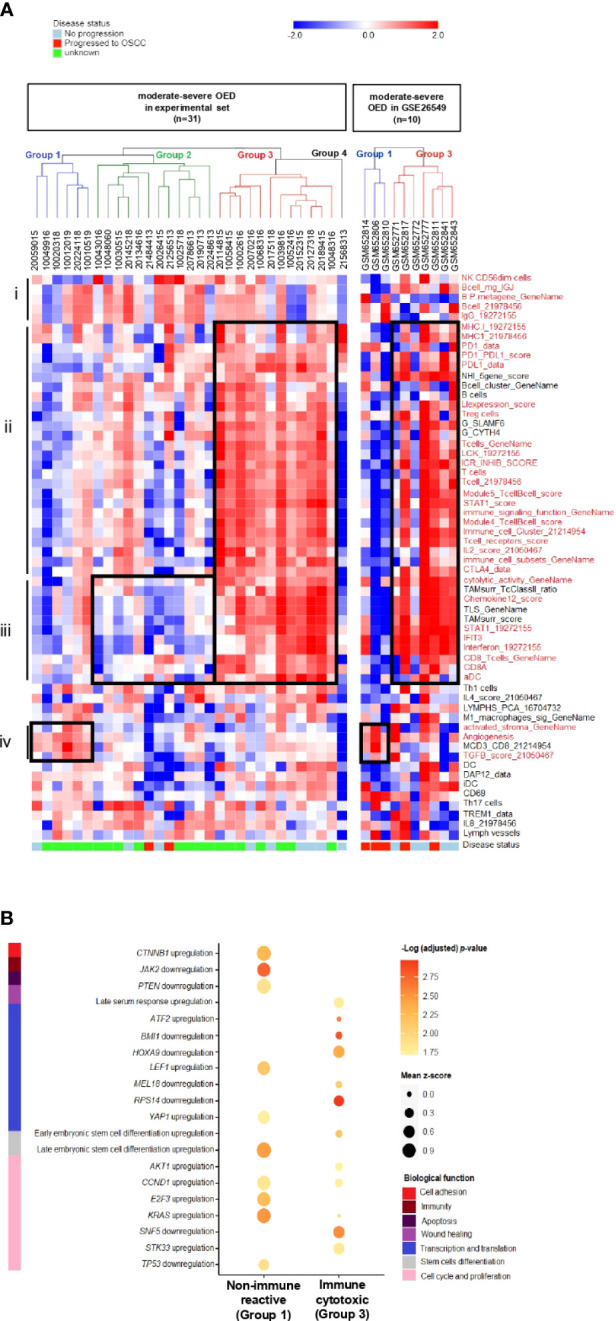
Discovery of distinct immune subtypes in moderate-severe OED and the identification of oncogenic pathways mediating immune evasion. **(A)** Immune signature enrichment clustered moderate-severe OED into four groups. Heatmap of unsupervised hierarchical clustering analysis of 58 immune signatures in 31 moderate-severe OED. These signatures were divided into 4 main sections: (i): Immune signatures containing NK cells and B cells. (ii): Immune signatures containing antigen presentation, T cell infiltration and immune checkpoints. (iii): Immune signatures related to CD8 T cell activation, interferon signaling and immune cytotoxicity (iv): Immune signatures related to oncogenic-promoting signals. Immune signatures in red are the representation of immune features described in section i to iv. Similarly, supervised hierarchical clustering of GSE26549 dataset showed that moderate and severe OED can be clustered into group 1 (non-immune reactive) and group 3 (immune cytotoxic) subtypes. **(B)** Enrichment plot of oncogenic signature gene sets in non-immune reactive and immune cytotoxic subtypes. The enrichment of oncogenic signature gene set was represented by the mean z-score of each subtype. Genes that promote immune evasion in cancer such as *CTNNB1*, *JAK2* and *PTEN* are detected in non-immune reactive subtype, and may be responsible for limiting T cell infiltration and cytotoxic response in this subtype. In addition, a relatively higher enrichment of oncogenic gene sets regulating cell cycle and proliferation was also detected in the non-immune reactive subtype when compared to the immune cytotoxic subtype.

To identify the immune subtype in high-risk OPMD, we subjected 31 moderate-severe OED samples from our RNA sequencing dataset to unsupervised hierarchical clustering analysis using the 58 immune signatures identified in the previous section. Notably, we found that our samples can be classified into four groups based on the cluster dendrogram branches (**Figure 6A**, left). Group 1 was designated as the non-immune reactive subtype. Samples in this group demonstrated enrichment of immune signatures related to tumor-promoting signals such as activated stroma, angiogenesis, and TGFB signaling, which may be involved in stromal modulation. Group 2 consisted of the non-cytotoxic subtype, as immune signatures involved in T cell activation, such as CD8 T cells, activated DC, interferon and cytolytic activity, were not enriched. Group 3 is known as the immune cytotoxic subtype, in which immune signatures representing T cell activation and immune-mediated cytotoxicity, along with those involved in antigen presentation and lymphocyte infiltration, are prominently enriched. Meanwhile, Group 4 consisted of only one sample and hence was not subjected to further analysis. A further comparison of the samples in the three immune subtypes with the matched H&E tissue sections showed that the non-immune reactive subtypes comprised moderate-severe OED demonstrating absence of immune infiltrate (n=2) and those with immune infiltrate (n=4; **Supplementary Figure 4**). It is possible that samples with immune infiltrate are found in non-immune-reactive subtype as we have not differentiated the immune cell types during the evaluation of immune infiltrate by H&E.

The immune cytotoxic subtype resembles an active immune surveillance environment owing to the enrichment of immune signatures related to lymphocyte infiltration and T cell activation. Furthermore, five of the nine samples with no disease progression were clustered in this group. However, the disease progression status of the other four samples in the immune cytotoxic subtype were unavailable. In contrast, the non-cytotoxic subtype lacks T cell activity (reduced enrichment in immune signatures representing CD8 T cells and cytolytic activity), and could be immune compromised because two samples that progressed to OSCC harbored this immune subtype. Only a small number of moderate-severe OED are non-immune reactive owing to the lack of T cell infiltration. A similar observation was obtained when the 58 immune signatures were applied to moderate-severe OED samples in the GSE26549 dataset. Interestingly, three of the five samples that progressed to OSCC harbored the non-immune reactive subtype (Group 1), whereas all five non-progressive OED harbored the immune cytotoxic subtype (Group 3) (**Figure 6A**, right).

Additionally, we identified potential oncogenic signals that may influence lymphocyte infiltration in the non-immune reactive subtype. Using the C6 oncogenic signature gene set, we found that the non-immune reactive subtype harbors an upregulation of *CTNNB1* and downregulation of *JAK2* and *PTEN* when compared to the immune cytotoxic subtype (**Figure 6B**). Dysregulation of these genes has been shown to limit T cell priming and immune cell recruitment in the tumor microenvironment (43), suggesting that these genes may be inhibiting T cell infiltration in the non-immune reactive subtype. Consistently, genes involved in cell cycle and proliferation (*AKT1, CCD1, E2F3* and *KRAS*) were upregulated in the non-immune reactive subtype. Meanwhile, the immune cytotoxic subtype showed downregulation of genes that regulate transcription and translation (*BMI1, HOXA9, MEL18* and *RPS14*). Our data suggests that immune inhibition in the non-immune reactive subtype is driven by *TGFB*, *CTNNB1, JAK2* and *PTEN* dysregulation, leading to upregulation of transcriptional regulation and cell proliferation, which may contribute to an increased risk of malignant transformation.

## Discussion

Immune reactivity is prominent in both epithelial and stromal compartments of OED (14, 15, 44, 45). Indeed, our study demonstrated that immune stimulation peaks at moderate-severe OED (high-risk OPMD) and maintains at high level in early stage OSCC when compared to benign hyperplastic lesions. This observation was similar to that reported in bronchial premalignant lesions, whereby the expression of immune-related genes was highly induced in high-grade lesions when compared to low-grade lesions (13). Conceivably, immune reactivity in moderate-severe OED exerts an anti-tumorigenic effect, since a large proportion of immune signatures related to immune surveillance were highly enriched compared to those representing immune suppressive mechanisms. Immune surveillance in moderate-severe OED resembled the immune responses observed in carcinogen-induced OPMD mice, which involve Th1 cells, CD8 T cells and pro-inflammatory cytokines (45, 46). Meanwhile, immune suppressive mechanisms detected in moderate-severe OED, such as Treg cells and immune checkpoints, have been indicated to play a role in immune evasion for premalignant disease progression (13, 26, 47–49). Due to the lack of patient follow-up information, the association of these immune suppressive mechanisms with OED progression was not determined. In comparison to moderate-severe OED, immune signatures associated with poor cancer prognosis were further induced in early stage OSCC, such as immune checkpoint, IL-8 and TREM1 We speculate that a continuous induction of these genes may lead to immune evasion. In early stage OSCC, the immune environment resembled an interferon-induced adaptive immune resistance (50), as immune signatures for interferon signaling, immune checkpoints, and those related to poor cancer prognosis were induced.

Many studies, including ours, have demonstrated the upregulation of PD-1/PD-L1 axis in OPMD (47, 48). The efficacy of anti-PD1 antibodies in preventing malignant transformation has been demonstrated in carcinogen-induced OPMD mice, where effector T cell activation, STAT1 activation, and secretion of IFNγ and granzyme B have been demonstrated (10, 51–55). Clinical trials using anti-PD1 antibodies for immunoprevention of OPMD are currently underway (NCT03692325, NCT04504552, NCT02882282, and NCT03603223). However, Levingston and colleagues demonstrated that the response to anti-PD1 was transient (54). Since the cost of anti-PD1 treatment is high, more work is needed to identify patients who would benefit from anti-PD-1/PD-L1 monoclonal antibody immunoprevention.

Previous transcriptomic studies indicated that OPMD can be classified into immune-rich and non-immune subtypes (56, 57)**. Our study further classified moderate-severe OED into three major immune subtypes:** immune cytotoxic, non-cytotoxic and non-immune reactive. Notably, T cell-mediated immune responses differed among the three immune subtypes, whereas B and NK cell responses appeared to be consistent. Therefore, H&E staining will not be able to distinguish high-risk OPMD samples into the three immune subtypes, and a panel of cytolytic T cells markers is needed.

A reduction in T cell infiltration in premalignant lesions is associated with disease progression (25, 58). Our limited patient follow-up information indicated that OED that progressed to OSCC may harbor non-cytotoxic or non-immune reactive subtypes, highlighting that the immune cytotoxic response plays a crucial role in influencing premalignant disease outcome. Additionally, oncogenic genes dysregulated in the non-immune reactive subtype are involved in stroma activation and immune cell exclusion. This may potentially generate a permissive environment for malignant transformation. For example, *TGFB* upregulation, *CTNNB1* activation, and loss of *PTEN* and *JAK2* reduce T cell priming and infiltration in cancer, leading to immune escape and immunotherapy resistance (43, 59–63). More recently, LOH in the chromosomal 9p arm involving the loss of *JAK2* and *PD-L1* was shown to affect immune responses in HNC and may promote immune evasion and foster the malignant transformation of OPMD (64). Hence, intervention to induce T cell infiltration or combination therapy targeting oncogenic genes involved in immunoediting may be a feasible approach to reactivate immune responses in the non-immune reactive subtype (65).

Our preliminary data suggested that oncogenic signals from epithelial cells can influence lymphocyte infiltration; hence, future studies should consider host epithelial-stroma interactions in affecting malignant transformation. Additionally, it is important to understand how T cells become dysfunctional over the course of tumorigenesis, as a subset of high-risk OPMD lacks T cell responses although lymphocytes are present. Therefore, further understanding of T cell differentiation and function during malignant transformation is warranted. Although mild OED has not been investigated in this study, future work should consider extending these immune subtypes to these low-risk OPMD but care should be taken in the selection of these samples as the majority of them are devoid of immune infiltration. It is worth mentioning that the current study has limited sample size and incomplete disease progression information. This may be overcome through research collaborations with different countries to increase cohort size. Furthermore, the immune profile in the current study was determined using predefined immune signatures reported in cancers, thus may have hindered the discovery of novel immune signatures that are unique in high-risk OPMD.

In conclusion, we have mapped out the immune responses underlying immune infiltrate in moderate-severe OED. Our immune profiling analysis suggested that three distinct immune subtypes are present in moderate-severe OED, which could potentially be used for risk stratification and the identification of therapeutic targets. The findings from this study will be followed up in an independent cohort with long-term patient follow-up information to investigate their influence on malignant transformation.

## Code availability

The R scripts used in this study is available at https://github.com/BernardKBLee/OPMD_proj/

## Data availability statement

RNA sequencing data from human OPMD FFPE tissue specimens can be found in the European Genome-phenome Archive (EGA) under EGA ID EGAS00001005520. The expression profile data analyzed in this study were obtained from the Gene Expression Omnibus (GEO) dataset GSE26549. All other data supporting the findings can also be obtained from the supplementary information files or from the corresponding author upon reasonable request.

## Ethics statement

The studies involving human participants were reviewed and approved by Medical Ethics Committee of the Faculty of Dentistry, University of Malaya [DF OS1624/0073(L)] and the Medical Research and Ethics Committee (MREC), Ministry of Health Malaysia [NMRR-16-1764-32566 (IIR)]. Written informed consent for participation was not required for this study in accordance with the national legislation and the institutional requirements.

## Author contributions

Study conception and design: CO, GT, NS, KL, SC, and CG. Collection of clinical specimens and patient information: CG, SL, TG, ZZ, RZ and HS. Development of methodology: CG, BKBL, and KL. Sample processing and data analysis: CG, BKBL, and BKWL. Pathological evaluation: SL, TG, ZZ, RZ and JY. Manuscript preparation: CG, KL, SC, and HA. Study supervision: NS, GT, HA, CO, SC, and KL. Manuscript revision: All authors. All authors contributed to the article and approved the submitted version.

## Funding

This study was funded by the Global Challenges Research Fund of the Medical Research Council, UK (MR/P024351/1), and internal funding from Cancer Research Malaysia.

## Acknowledgments

We thank the Ong Heng Tiang & Ong Sek Pek Foundation for sponsoring a scholarship to CG. We thank Mr. Chee Yong Fong for technical support in sample processing, Ms. Sherlly Lim for the preparation of mIF images, and Ms. Hui Shi Saw for assisting with bioinformatic analysis. The authors would also like to thank the Director General of Health Malaysia for permission to publish this paper.

## Conflict of interest

The authors declare that the research was conducted in the absence of any commercial or financial relationships that could be construed as a potential conflict of interest.

## Publisher’s Note

All claims expressed in this article are solely those of the authors and do not necessarily represent those of their affiliated organizations, or those of the publisher, the editors and the reviewers. Any product that may be evaluated in this article, or claim that may be made by its manufacturer, is not guaranteed or endorsed by the publisher.
