# Supplementary material for: Transcriptional analysis highlights three distinct immune profiles of high-risk oral epithelial dysplasia
Source: Front Immunol. 2022 Sep 2;13:954567. doi: 10.3389/fimmu.2022.954567 (PMC9479061; doi:10.3389/fimmu.2022.954567)

Supplementary Figure 1: Identification of gene modules with specific co-expression trends in moderate-severe OED and early stage OSCC. (a) Thirty gene modules were derived from WGCNA. Dendrogram demonstrating the hierarchical clustering of 18938 genes based on topological overlap matrix. Thirty co-expression gene modules were identified and were assigned with different module colours at the bottom. **(b) Three gene co-expression trends and the associated gene modules.** Nine of the thirty gene modules showed significant differences in ssGSEA scores when compared across FEP, moderate-severe OED and early stage OSCC($p<0.05$). These nine gene modules were further classified into the three co-expression trends as following: (i) Increased in moderate-severe OED and early stage OSCC over FEP but with no change between moderate-severe OED and early stage OSCC (ii) Progressive gain of expression (iii) Progressive loss of expression.

(a)

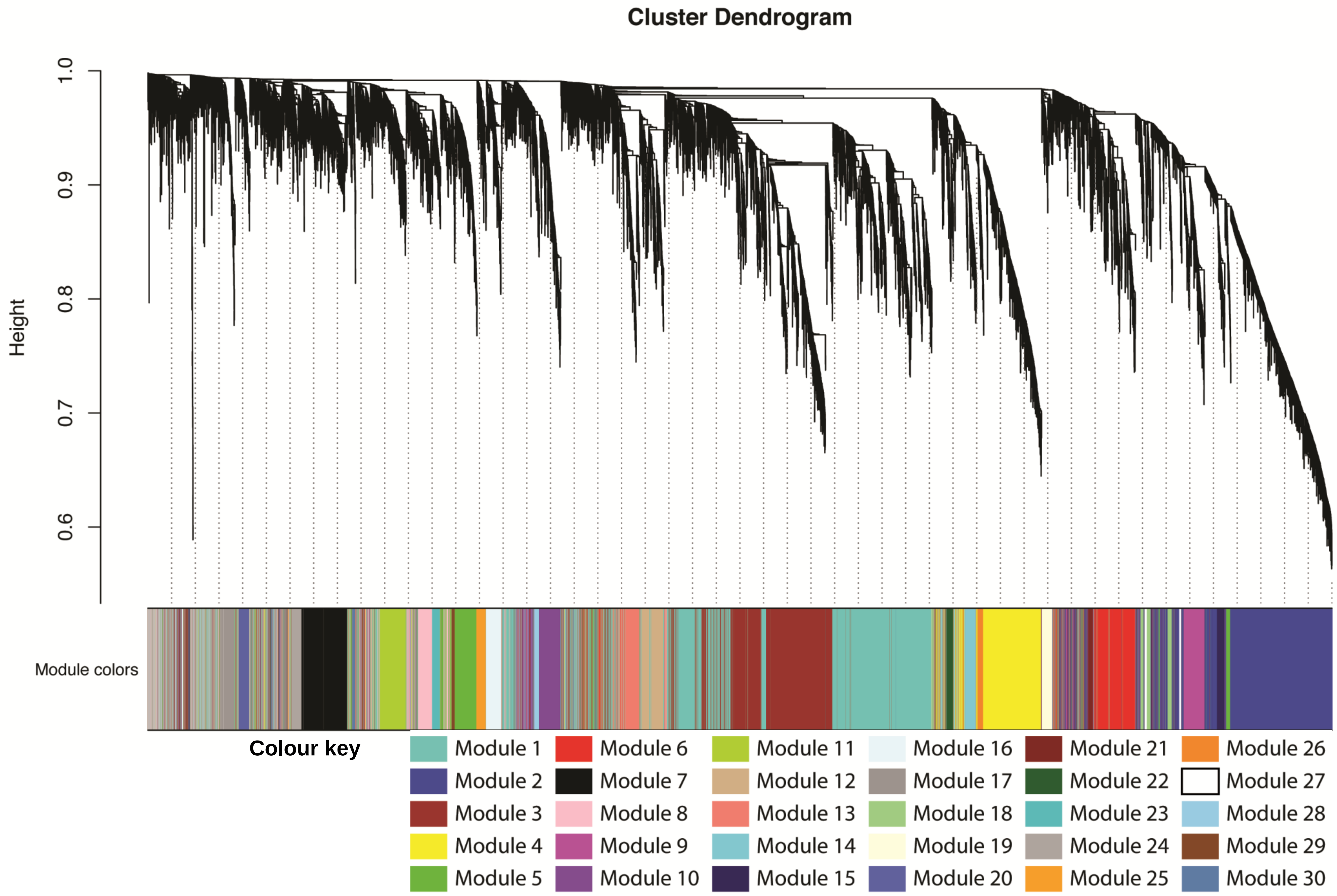

(b)

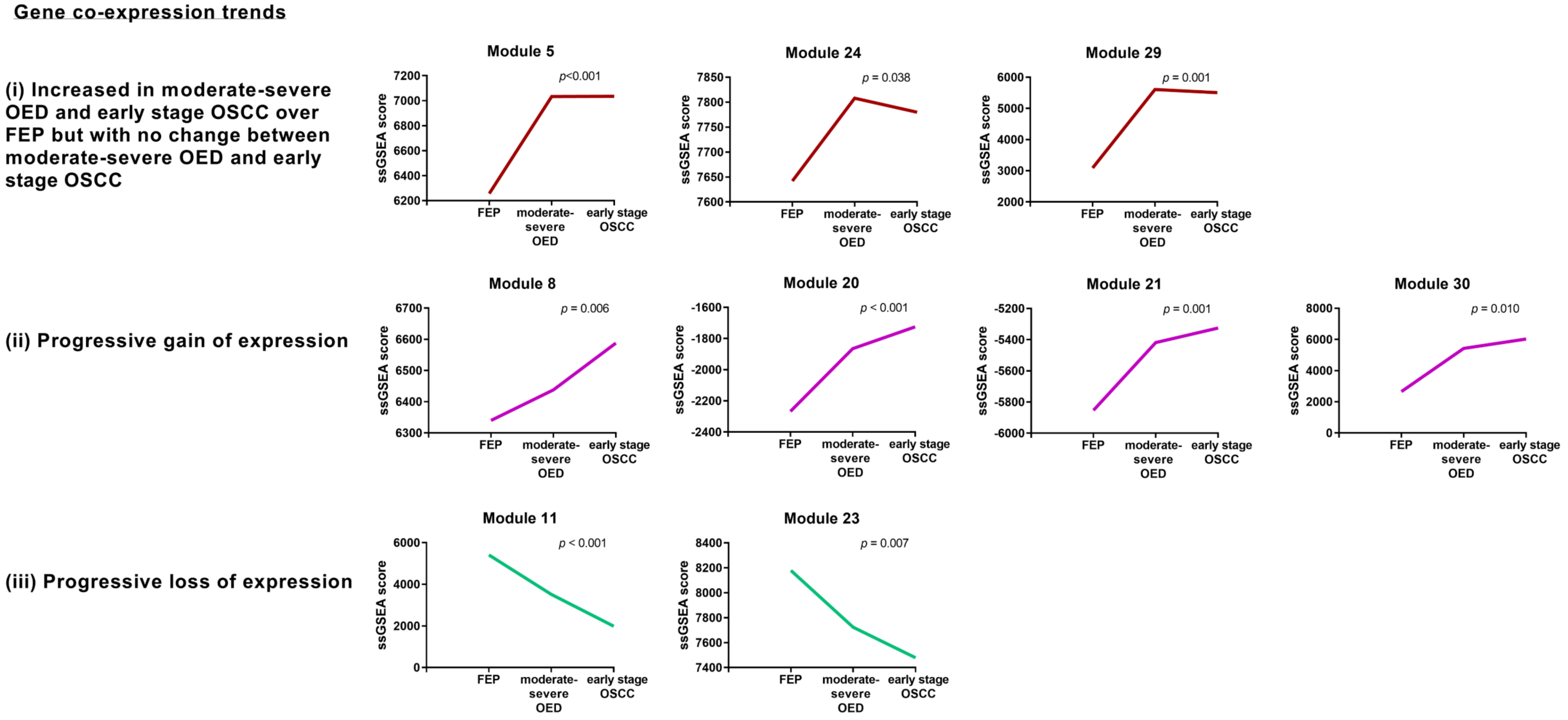

Supplement: Supplementary file 1 [file DataSheet_1.pdf]
