# Supplementary material for: Transcriptional analysis highlights three distinct immune profiles of high-risk oral epithelial dysplasia
Source: Front Immunol. 2022 Sep 2;13:954567. doi: 10.3389/fimmu.2022.954567 (PMC9479061; doi:10.3389/fimmu.2022.954567)

Supplementary figure 2: Immune signatures enrichment in moderate-severe OED and early stage OSCC. A total of 58 immune signatures demonstrated differential enrichment in moderate-severe OED and early stage OSCC when compared to FEP (FDR<0.1). The y-axis of the violin plot represents z-scores of the immune signatures derived from ssGSEA analysis.

(a) Immune signatures demonstrating differential enrichments in moderate-severe OED in comparison to FEP

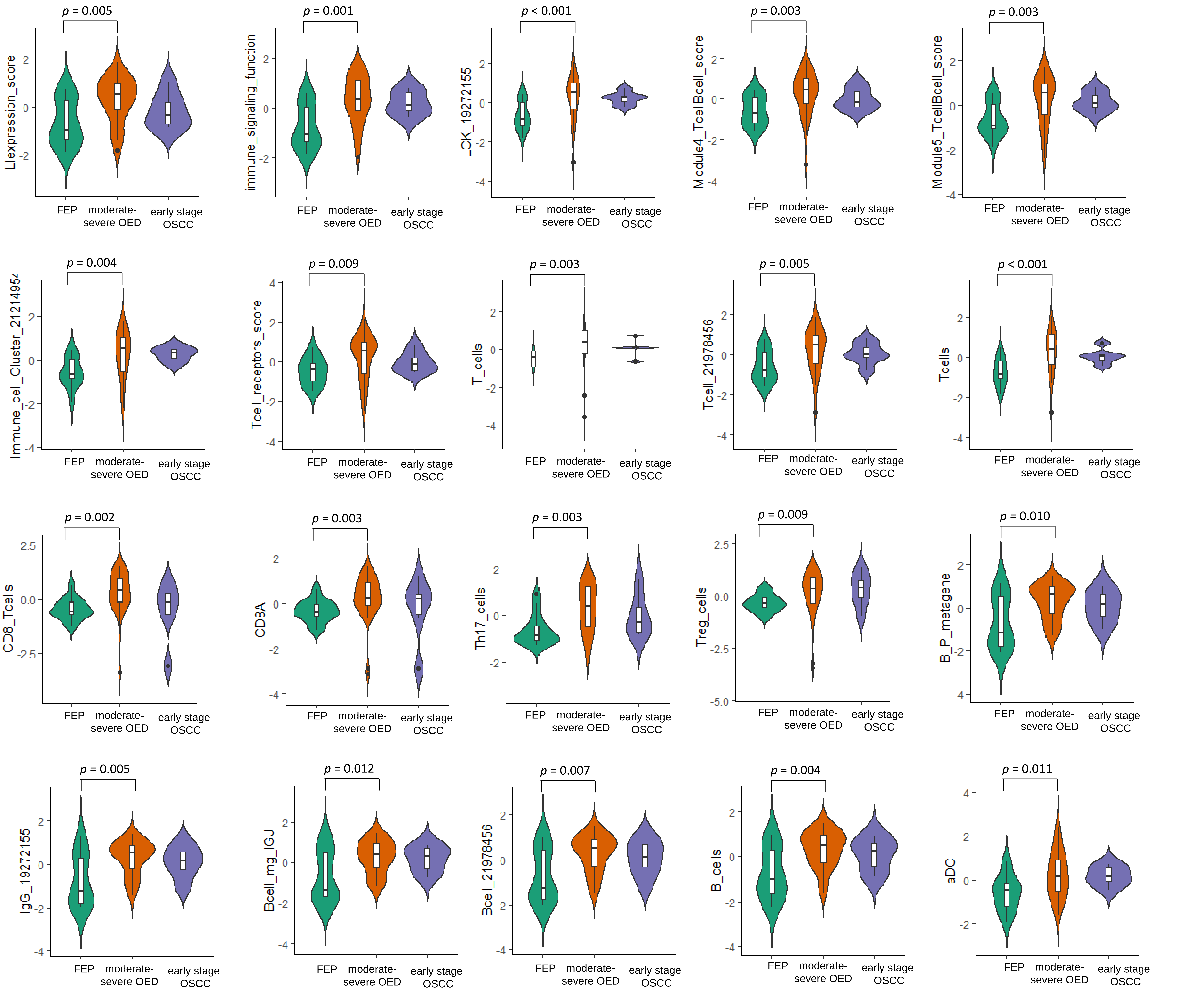

(a)) Immune signatures demonstrating differential enrichments in moderate-severe OED in comparison to FEP (continue)

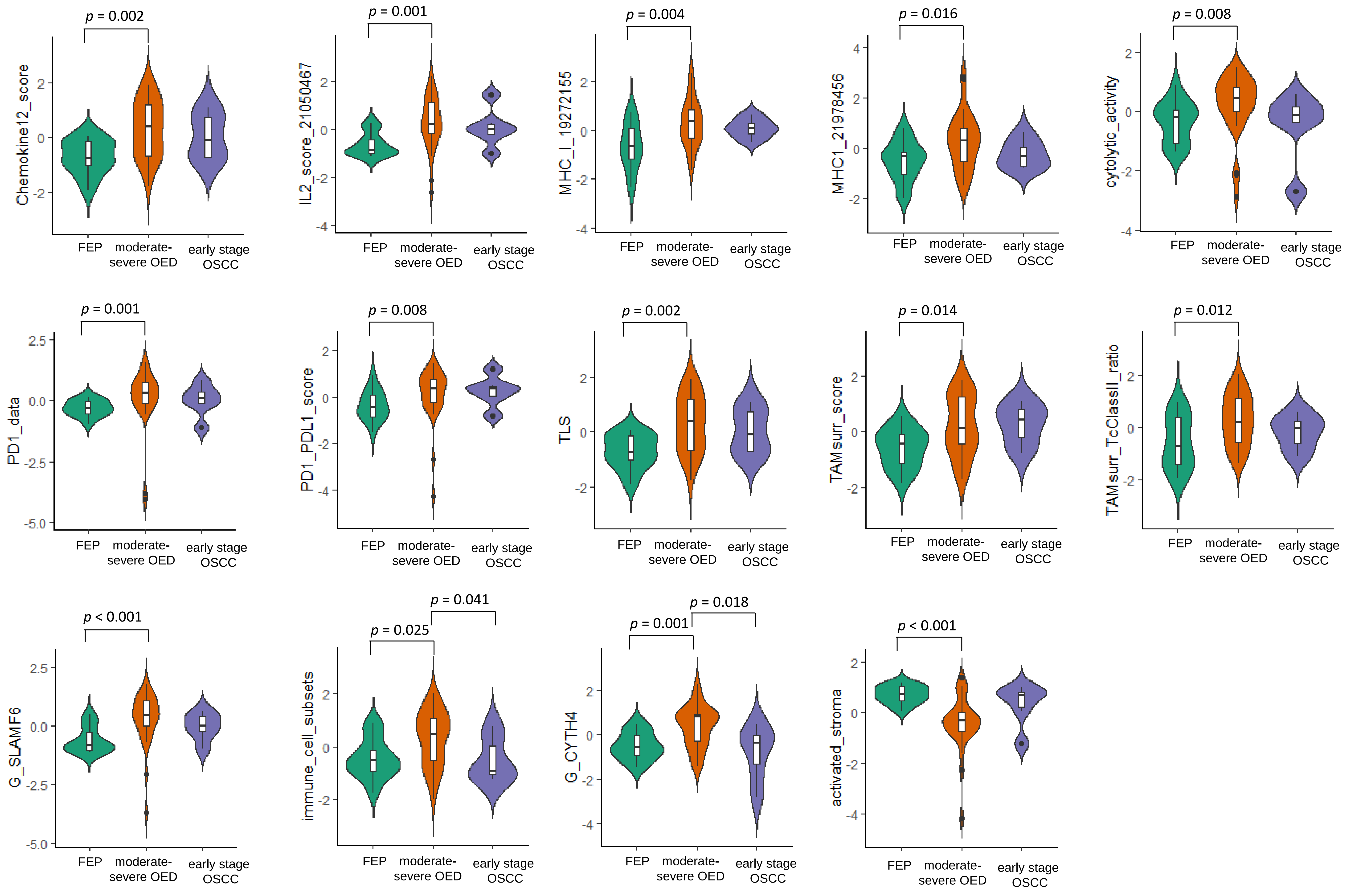

(b) Immune signatures demonstrating differential enrichments in moderate-severe and early stage OSCC OED in comparison to FEP

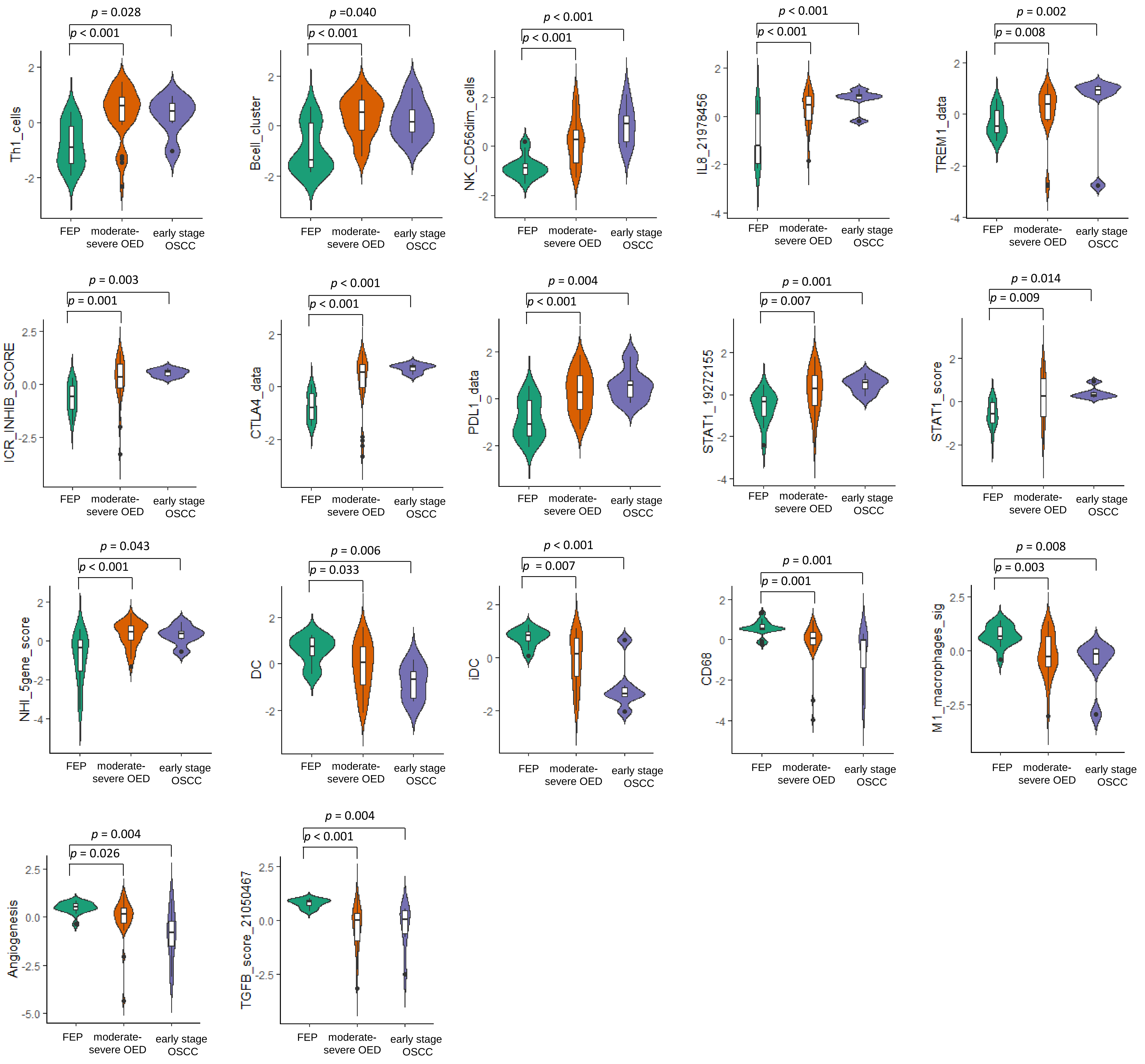

(c) Immune signatures demonstrating differential enrichments in early stage OSCC in comparison to FEP

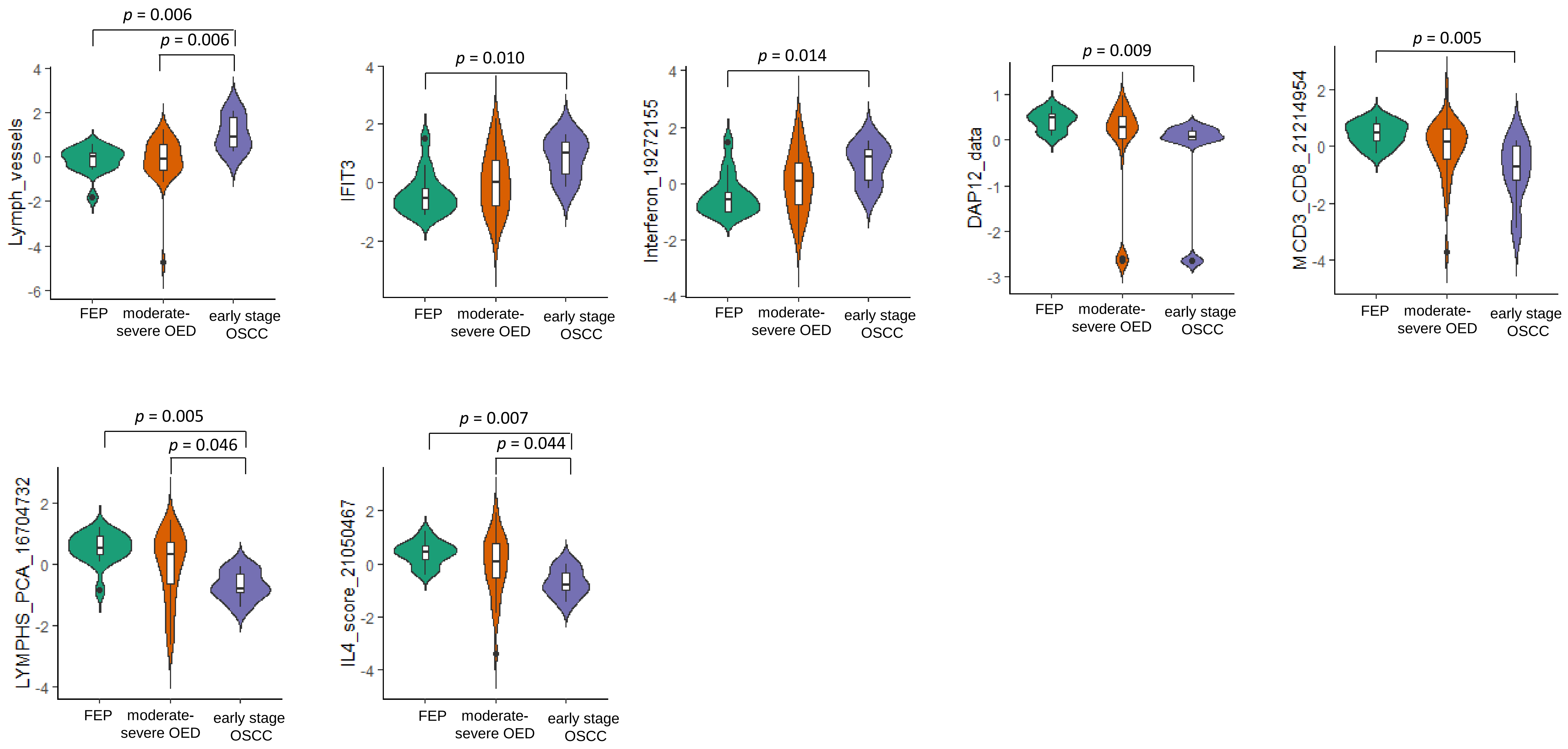

Supplement: Supplementary file 2 [file DataSheet_2.pdf]
