# Supplementary material for: Transcriptional analysis highlights three distinct immune profiles of high-risk oral epithelial dysplasia
Source: Front Immunol. 2022 Sep 2;13:954567. doi: 10.3389/fimmu.2022.954567 (PMC9479061; doi:10.3389/fimmu.2022.954567)

**Supplementary figure 3: Immune signatures enriched in early stage tumours from our RNA sequencing dataset is validated with HNC TCGA dataset.** Immune signatures enriched in early stage OSCC were validated by subjecting 489 HNC samples from TCGA dataset to ssGSEA. The enrichment of these immune signatures in the early stage tumours from HNC TCGA dataset (green) and our RNA sequencing dataset (blue) were determined by comparing to the respective control samples in each dataset. Violin plots showing the median (black solid line), first quartile and third quartile horizontal lines flanking the median) value for each group.

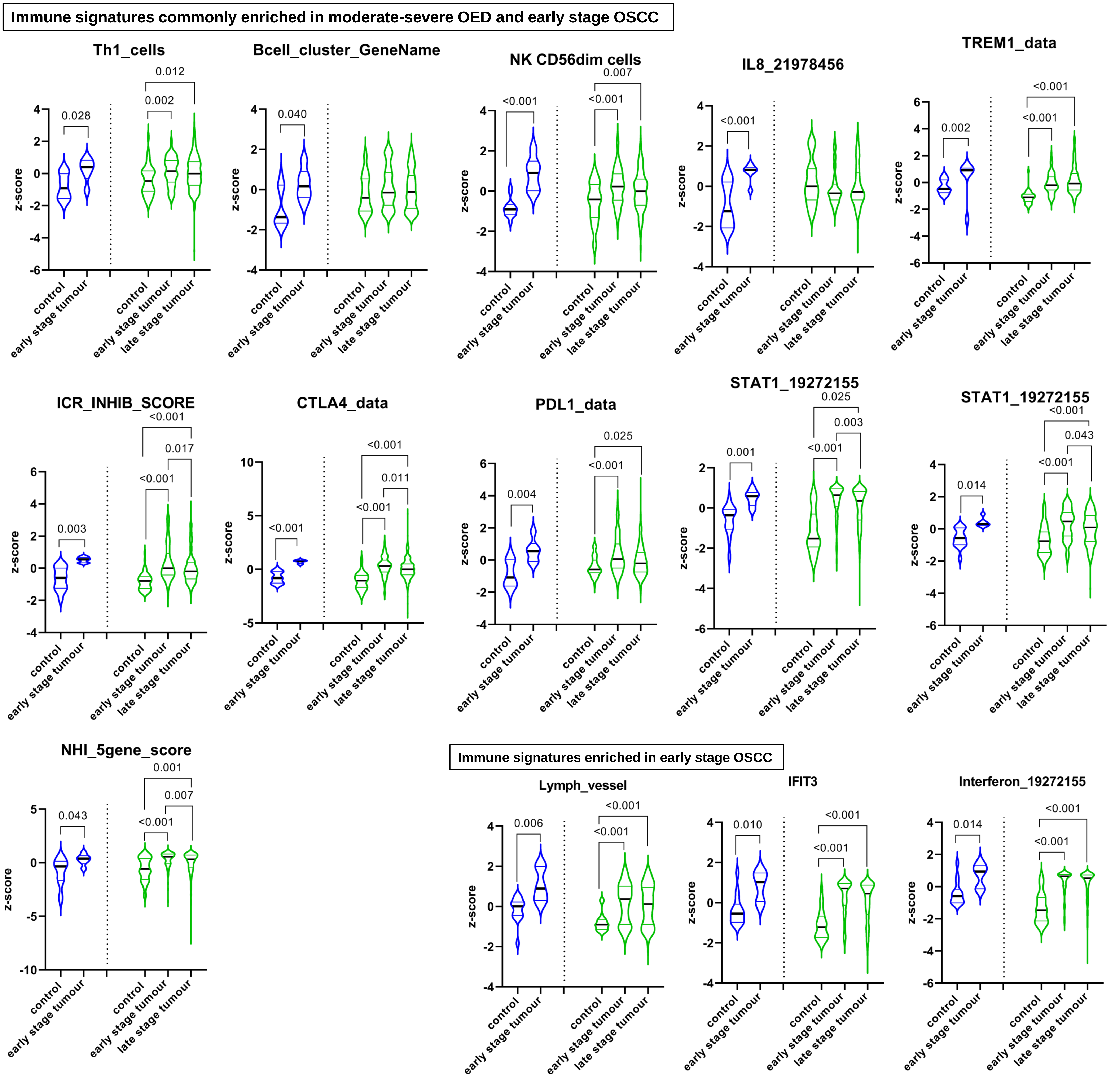

Supplement: Supplementary file 3 [file DataSheet_3.pdf]
