# Supplementary material for: Transcriptional analysis highlights three distinct immune profiles of high-risk oral epithelial dysplasia
Source: Front Immunol. 2022 Sep 2;13:954567. doi: 10.3389/fimmu.2022.954567 (PMC9479061; doi:10.3389/fimmu.2022.954567)

Supplementary figure 4: Comparison of immune subtypes determined by RNA sequencing and the immune infiltrate observed on H&E-stained sections of matched samples. (a) Distribution of samples according to immune subtype and pattern of immune infiltrate. (b)Representative H&E images of samples used in RNA sequencing. Immune cells appeared as small round purple dots in the H&E-stained sections. Lymphocytes infiltrated into the epithelial are indicated by black triangle, while macrophages are marked by yellow arrow.

(a)

Subtypes by RNA sequencing	Immune infiltrate by H&E			
	Infiltrated from stroma into epithelial compartment	Present in stroma	Absence of immune infiltrate	Total
Non-immune reactive	4	0	2	6
Non-cytotoxic	8	4	0	12
Immune cytotoxic	12	0	0	12
Others	0	0	1	1

(b)

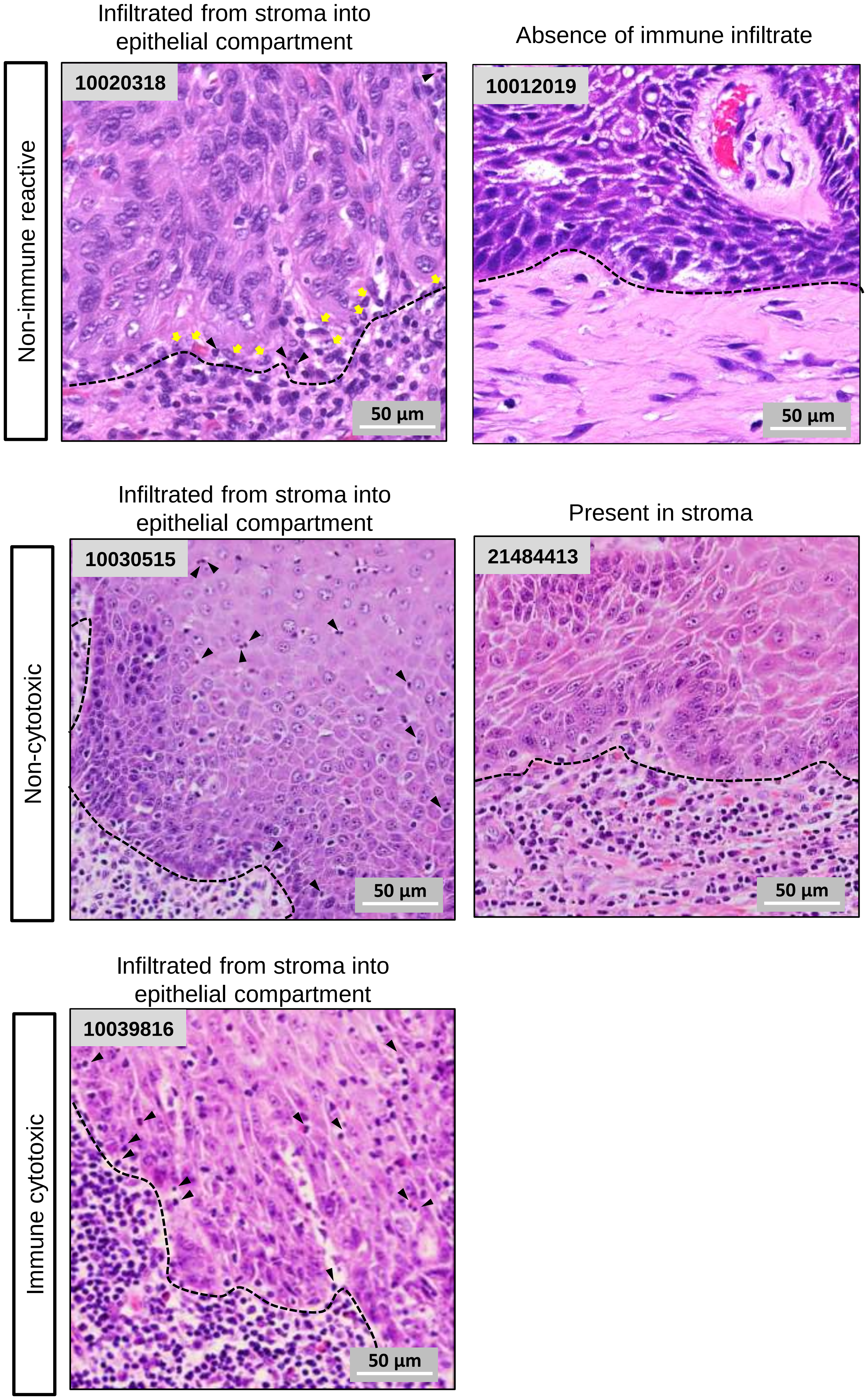

Supplement: Supplementary file 4 [file DataSheet_4.pdf]
